# MAD2B promotes podocyte injury through regulating Numb-dependent Notch 1 pathway in diabetic nephropathy

**DOI:** 10.7150/ijbs.68977

**Published:** 2022-02-21

**Authors:** Meng-Ran Li, Chun-Tao Lei, Hui Tang, Xing-Jie Yin, Zhe Hao, Yang Qiu, Ya-Ru Xie, Jie-Yu Zeng, Hua Su, Chun Zhang

**Affiliations:** Department of Nephrology, Union Hospital, Tongji Medical College, Huazhong University of Science and Technology, Wuhan 430022, China.

**Keywords:** podocyte injury, diabetic nephropathy, MAD2B, Numb, Notch 1 pathway

## Abstract

**Rationale:** Recent studies have demonstrated that the loss of podocyte is a critical event in diabetic nephropathy (DN). Previously, our group have found that the mitotic arrest deficient protein MAD2B was involved in high glucose (HG)-induced podocyte injury by regulating APC/C activity. However, the exact mechanism of MAD2B implicated in podocyte injury is still lacking.

**Methods:** The experiments were conducted by using kidney tissues from streptozotocin (STZ) induced diabetic mice with or without podocyte-specific deletion of MAD2B and the cultured podocytes exposed to different treatments. Glomerular pathological injury was evaluated by periodic acid-Schiff staining and transmission electron microscopy. The endogenous interaction between MAD2B and Numb was discovered by yeast two-hybrid analysis and co-immunoprecipitation assay. The expressions of MAD2B, Numb and related pathway were detected by western blot, immunochemistry and immunofluorescence.

**Results:** The present study revealed that MAD2B was upregulated in diabetic glomeruli and cultured podocytes under hyperglycemic conditions. Podocyte-specific deletion of MAD2B alleviated podocyte injury and renal function deterioration in mice of diabetic nephropathy. Afterwards, MAD2B was found to interact with Numb, which was downregulated in diabetic glomeruli and HG-stimulated cultured podocytes. Interestingly, MAD2B genetic deletion could partly reverse the decline of Numb in podocytes exposed to HG and in diabetic mice, and the expressions of Numb downstream molecules such as NICD and Hes-1 were decreased accordingly. In addition, overexpression of Numb ameliorated HG-induced podocyte injury.

**Conclusions:** The present findings suggest that upregulated MAD2B expression contributes to Numb depletion and activation of Notch 1 signaling pathway, which ultimately leads to podocyte injury during DN progression.

## Introduction

Diabetic nephropathy (DN) is one of the major microvascular complications in diabetes mellitus and the leading cause of chronic and end-stage renal disease (ESRD) in developed countries 1. Its clinical features include gradual increase in proteinuria, blood pressure, and risk of cardiovascular disease, and decrease in glomerular filtration rate (GFR) 2. Podocytes, highly differentiated glomerular epithelial cells, are essential for the maintenance of glomerular filtration barrier 3. Growing evidences indicate that podocytes loss is a critical factor in DN development 4. However, effective treatments for DN to prevent or reduce podocyte loss are still lacking currently 5, 6. Therefore, clarifying the underlying mechanisms of podocyte loss and identifying the potential therapeutic targets for DN have clinical importance.

Mitotic spindle assembly check point protein 2 (MAD2B), also termed MAD2L2 and REV7, is an inhibitor of anaphase-promoting complex/cyclosome (APC/C) and a small subunit of DNA polymerase-ξ, widely expressed in the tissues of human brain, liver, breast, colon and so on. MAD2B regulates the cell cycle by directly binding to both CDC20 and CDH1, two APC/C activator subunits, thus inhibiting the activation of APC 7. In addition, MAD2B is involved in translesion DNA synthesis and nonhomologous end joining 8. Our previous study first confirmed that MAD2B is widely expressed in mouse kidney and in various kidney resident cells, including glomerular mesangial cells, tubular epithelial cells and podocytes. We subsequently verified that the expression of MAD2B was elevated not only in the glomeruli of DN patients and db/db mice, but also in HG stimulated podocytes, followed by cyclin B1 and Skp2 accumulation 9. However, the exact mechanism how MAD2B regulates podocyte injury in DN remains largely unknown.

Notch1 is a membrane receptor of Notch signaling, which plays important roles in cell-fate decisions and differentiations during the embryonic development of kidney and then becomes mostly silent after kidney maturation 10. Recent studies have indicated that Notch1 is reactivated in podocytes under diabetic conditions resulting in its disfunction by inducing epithelial-mesenchymal transition (EMT), apoptosis, autophagy decrease, inflammation and loss in numbers of podocytes 11-14. Our previous experiments have found that HG induced reactivation of Notch 1 signaling pathway can be inhibited by silencing MAD2B in podocytes. Numb is originally identified as an inhibitor of Notch signaling 15, which is a cargo-selective endocytic adaptor widely expressed in different types of mammal cells 16. It is reported that the protein expression of Numb is decreased in human DN tissues and HG-stimulated human glomerular endothelial cells (RGEC), while Notch1 expression is in the opposite. Numb is then proven to delay EMT progression in DN by negatively regulating Notch signaling pathway 17. However, little is known about the role of Numb in podocyte injury and its exact relationship with MAD2B.

In this study, we found that the level of MAD2B was increased in podocytes under hyperglycemia *in vitro* and *in vivo*, while Numb was declined. Podocyte-specific deletion of MAD2B alleviated podocyte injury and proteinuria in STZ-induced mouse DN model. We further observed that MAD2B interacted with Numb, and inhibited the role of Numb in negatively regulating Notch1 signaling under diabetic conditions. Meanwhile, overexpression of Numb attenuated podocyte damage *in vitro*. Mechanistically, MAD2B negatively regulated Numb and activated Notch1 signaling accordingly, ultimately contributing to podocyte injury in DN. Our findings provide new insights into the exact mechanism how MAD2B mediates podocyte injury in DN.

## Methods

### Animal study

All animal experiments were approved by the Ethics Committee of Huazhong University of Science and Technology. Mice were treated humanely and all procedures were conducted in accordance with the guidelines for the use and care of laboratory animals of the National Institutes of Health. Different groups were allocated in a randomized manner and animals were raised under standard laboratory conditions with a twelve-hour light and dark cycle and allowed free access to water and standard chow diet. C57BL/6J male mice (7-8-week-old) were obtained from Charles River (Beijing, China).

### Generation of podocyte-specific MAD2B knockout mice

MAD2B^fl/fl^ mice (C57BL/6J) were generated by Shanghai Southern Model Biotechnology Development Company, Ltd. (Shanghai, China). In these mice, MAD2B exon 3 and 4 were flanked by loxP sequences. MAD2B^fl/fl^ mice (C57BL/6J) were hybridized with mice expressing Cre recombinase (Cre) under the control of the podocin promoter (B6. Cg-Tg [NPHS2-cre] 295Lbh/J; Stock No.008205, Jackson Laboratory) to generate podocyte-specific MAD2B knockout mice (Nphs2-Cre^+^/ MAD2B^fl/fl^ mice). Mouse genotyping was confirmed by tail DNA and PCR at 4 weeks of age with primers: flox genotyping primers (forward 5'-TCTTCCCTTAGATTGGGTTTCTC-3'; reverse 5'-GCACGAATAGGACAAACAGCAA-3') and Cre genotyping primers (forward 5'-GCGCTGCTGCTCCAG-3'; reverse 5'- CGGTTATTCAACTTGCACCA-3'). Wild type presents only a 276 bp band; homozygous (MAD2B^fl/fl^) presents only a 306 bp band; heterozygous (MAD2B^fl/+^) presents both bands. Cre positive (Cre^+^) presents at 100 bp, but Cre negative (Cre^-^) has no band.

### Streptozotocin-induced DN in mice

Diabetes was induced by an intraperitoneal injection of high-dose streptozotocin (STZ, 150 mg/kg, BOSTER, Wuhan, China) as described in our previous studies 18. Before STZ injection, mice were starving for 12 h to induce insulin deficiency. After one week, blood glucose levels were measured and the mice with glucose levels above 16.7 mmol/L were included in the following experiment. All mice had free access to food and water, and were maintained for 12 weeks. Serum samples and 24-h urine samples were collected for biochemical analysis. Kidney tissues were harvested for histopathological and protein analysis.

### Glomeruli isolation

Mouse glomeruli were isolated by using a modified method as described previously 19. Mice were anesthetized and perfused slowly with inactivated Dynabeads (2×10^7^ beads/mouse, M-450 Tosylactivated, Invitrogen, Carlsbad, CA, USA) diluted in phosphate-buffered saline (PBS) through renal intravenous injection. Kidneys were removed and cortex, medulla, and papilla were separated by dissection. Cortex was minced into 1 mm^3^ pieces and digested in collagenase A buffer (Sigma-Aldrich, St. Louis, MO, USA) at 37 °C for 15 min with gentle agitation. Then, the digested tissue was pressed through a 100 μm cell strainer and flushed by ice-cold PBS. Cell suspension was centrifuged at 1500 rpm at 4 °C for 5 min to collect the pellet. The pellet was resuspended in PBS, and the glomeruli containing Dynabeads were gathered using a magnetic particle concentrator and washed at least 3 times with PBS. The purity of glomeruli was estimated by using a light microscope to inspect glomeruli suspensions on glass slides.

### Biochemical analysis of serum and urine samples

Serum albumin, serum creatinine, blood urea nitrogen and urine creatinine levels were measured by enzymatic assays using an auto-chemistry analyzer. Urine albumin was detected using an ELISA kit (ab108792, Abcam, Cambridge, MA, USA) according to the manufacturer's instructions. The urine albumin excretion rate was expressed as the ratio of albumin to creatinine.

### Immunohistochemistry (IHC)

Kidney tissues were transferred to 4% paraformaldehyde and fixed at 4 °C overnight. 4-μm paraffin embedded sections were deparaffinized and rehydrated for immunohistochemical staining. The sections were then incubated with the primary antibody for MAD2B (1:100, ab180579, Abcam, Cambridge, MA, USA), Numb (1:200, #2756, Cell Signaling Technology, Danvers, MA, USA) and WT1 (1:100, ab88901, Abcam, Cambridge, MA, USA) overnight at 4 °C. After being stained with hematoxylin, the sections were observed under a light microscope. We calculated the positive stained areas per glomerulus using Image-Pro Plus 6.0 software.

### Immunofluorescence staining (IF)

Frozen kidney tissues and cells were transferred to 4% paraformaldehyde and fixed at 4 °C overnight. Cells and 4-μm tissue sections were permeabilized with 0.3% Triton X-100 for 10 min and blocked with 5% donkey serum for 1 h. They were then incubated with the following primary antibodies: anti-MAD2B (1:200, ab180579, Abcam, Cambridge, MA, USA), Numb (1:50, sc-136554, Santa Cruz, CA, USA), anti-Synaptopodin (1:200, #163004, Synaptic Systems, Gottingen, Germany), and anti-Nephrin (1:100, AF3159, R&D, Minneapolis, MN, USA) overnight at 4 °C. Alexa Fluor 488 IgG and Alexa Fluor 594 IgG (1:200, Invitrogen, Carlsbad, CA, USA) were used as secondary antibodies. Nucleus was counterstained with Hoechst (Beyotime Biotechnology, Shanghai, China). Sections were observed under fluorescence microscope and images were captured at identical microscopic settings.

### Histopathological staining

Periodic acid-Schiff (PAS) staining of 4-μm paraffin sections was performed to evaluate the mesangial expansion in the glomeruli. At least 10 glomeruli per section were measured in a blinded fashion and the positive area per glomerulus area was quantified using Image-Pro Plus 6.0 software (Media Cybernetics, Rockville, MD, USA).

### Transmission electron microscopy (TEM)

Electron microscopic sample handling and detection were performed using an electron microscopic core as described earlier 20. To determine the GBM thickness, foot process width, and the number of foot processes per micrometer of GBM, TEM images were analyzed using Image J software (NIH, Bethesda, MD, USA).

### Yeast two-hybrid analysis

Yeast two-hybrid analysis and filter lift assays were performed as described previously 21. In brief, yeast cells were transfected with a bait plasmid carrying the MAD2B coding sequence, and then a yeast two-hybrid screen of a fetal human renal cDNA library was performed. We retested nine prey plasmids for their positive phenotype and conducted yeast mating experiments to exclude the false positive clones. Finally, three clones were β-galactosidase-positive, all of which were Numb (GenBank no. NM_001320114.1).

### Cell culture, treatment and RNA interference

A conditionally immortalized human podocyte cell line was cultured and maintained as described previously 22. In brief, podocytes were cultured at 33 °C for proliferation in RPMI 1640 medium supplemented with 10% fetal bovine serum (FBS), 100 U/mL penicillin, and 100 U/mL streptomycin. Upon reaching 70% confluence, podocytes were maintained at 37 °C for 10-14 days to induce differentiation. Differentiated podocytes were exposed to high glucose (HG, at a final concentration of 35 mmol/L in culture medium) for certain times to mimic the diabetic environment. Medium contained 5.6 mmol/L glucose was used as control and mannitol was added in the medium as the osmotic pressure control for HG. A lentivirus vector harboring a short-hairpin RNA (shRNA) sequence targeting MAD2B was synthesized by Jikai Gene (Shanghai, China), and scrambled shRNA was used as a control. Adenovirus vectors containing the DNA target sequence for Numb were obtained from Vigene Biosciences (Shandong, China). Podocytes were transfected with lentivirus or adenovirus to interfere with MAD2B expression or induce Numb overexpression according to the manufacturer's instructions.

### Western blotting

The total protein of cultured cells and isolated renal glomeruli were extracted with RIPA lysis buffer (Beyotime, Shanghai, China). The total protein lysates were measured by a BCA protein assay kit (Beyotime). After being boiled for 5 min at 95 °C in SDS protein loading buffer, proteins were separated by SDS-PAGE and transferred onto PVDF membranes (Millipore Corp., Bedford, MA, USA). The membranes were blocked in 5% non-fat milk for 1 h at room temperature and then incubated with primary antibodies overnight at 4 °C. The following primary antibodies against the following targets were used in this study: MAD2B (1:1000, ab180579, Abcam), Numb (1:1500, ab4147, Abcam), NICD (1:1000, ab52627, Abcam), Hes-1 (1:1000, ab71559, Abcam), Desmin (1:1000, BS1712, Bioworld Technology, Louis Park, MN, USA), MDM2 (1:1000, ab16895, Abcam), active β-catenin (1:1000, #4270, Cell Signaling Technology, Danvers, MA, USA), β-actin (1:10000, sc-47778, Santa Cruz, CA, USA) and α-tubulin (1:3000, Protein Tech Group, Chicago, IL, USA). Finally, the membranes were incubated with horseradish secondary antibodies and detected by an ECL system (Thermo). The densitometric analysis of western blot images was measured by ImageJ software (National Institutes of Health).

### Co-immunoprecipitation (Co-IP)

The whole cell lysates were prepared just as described above. Then equal amounts (1 mg) of total protein samples from three groups (Ctrl, HG and IgG) were incubated with 1 μg primary antibody overnight at 4 °C on a rotating device. The next day, 30 μl Protein A/G PLUS-Agarose (Santa Cruz Biotechnology, Santa Cruz, CA, USA) that had been washed with ice-cold PBS and centrifuged at 2,500 rpm for 5 min at 4 °C to remove supernatant, was then added to the mixture and rotated for 1 h at 4 °C. The pellets were collected by centrifugation at 2,500 rpm for 5 min at 4 °C and washed 3 times with 1.0 ml PBS for 5 min each time on a rotating device. Then the pellets were resuspended in 2 × SDS sample buffer and boiled in 98 °C for 5 min. The immunoprecipitated proteins were detected by western blotting analysis. The following primary antibodies were used for Co-IP in our present study: rabbit anti-MAD2B antibody (ab180579, Abcam), goat anti-Numb antibody (ab4147, Abcam), and horseradish peroxidase labeled donkey anti goat immunoglobulin G (A0181, Beyotime).

### F-actin staining

To assess podocyte cytoskeleton arrangement, podocytes cultured on glass coverslips were washed using PBS, fixed using 4% paraformaldehyde solution for 10 min and incubated with rhodamine-phalloidin (Sigma-Aldrich, St Louis, MO, USA) for 30 min before observation, which was diluted in PBS containing 10% donkey serum and 0.3% Triton X-100. One hundred cells were counted to calculate the ratio of cells retaining distinct F-actin fibers in different groups.

### Statistics

Data were expressed as the mean ± SEM. Analysis and graphing were performed using GraphPad software (GraphPad, San Diego, CA, USA). Student's test was used for comparison between two groups. *P* < 0.05 was considered statistically significant.

### Study approval

All animal experimental procedures performed in this study were approved by the Ethics Committee of Huazhong University of Science and Technology. Animal experiments were carried out according to the guidelines for use and care of laboratory animals of the National Institutes of Health and ratified by the Animal Care and Use Committee of Tongji Medical College.

## Results

### MAD2B was upregulated in the kidney from STZ-induced diabetic mice and in podocytes under high glucose condition

**Figure 1:**
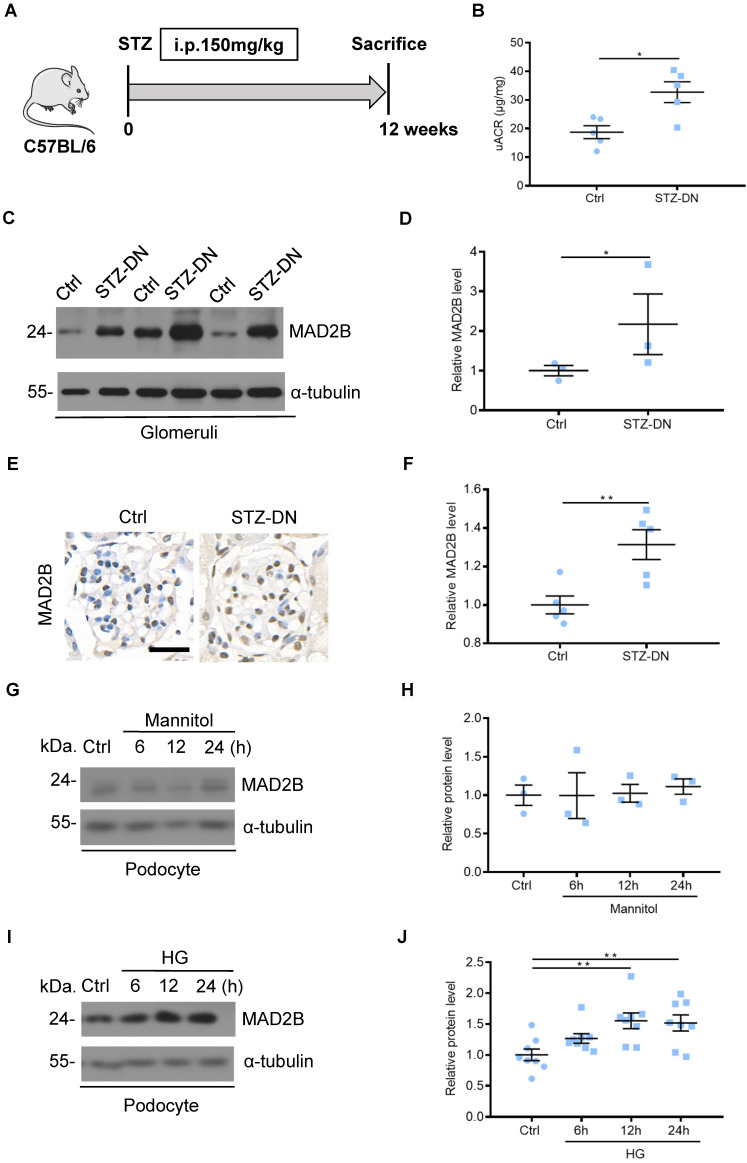
** MAD2B was upregulated in the kidney from streptozotocin (STZ)-induced diabetic mice and in podocytes under high glucose (HG) condition. (A)** A schematic diagram showing the method of building STZ-induced diabetic nephropathy (DN) model. Eight weeks old C57BL/6J mice were given intraperitoneal injection of 150 mg/kg streptozotocin and sacrificed after 12 weeks. **(B)** Urinary albumin to creatinine ratio (uACR) in different groups of mice (n=5). **(C and D)** Representative western blot and quantification of MAD2B expression in isolated glomeruli from control and DN mice (n = 3). *P<0.05. **(E and F)** Representative immunohistochemical images and quantification of glomerular MAD2B expression in STZ-induced diabetic mice (n = 5). Scale bar: 25 µm. **P<0.01. **(G and H)** Representative western blot and quantification data presenting the relative MAD2B and Numb protein levels in podocytes exposed to 29.4 mM mannitol plus 5.6 mmol/L D-glucose for indicated time. (n = 3), *P*>0.05. **(I and J)** Representative western blot and quantification of MAD2B expression in podocytes exposed to 35 mM HG for indicated time (n = 8). **P<0.01. All data were expressed as mean ± SEM.

To detect MAD2B protein levels in DN, C57BL/6J mice were induced to develop a DN model with STZ treatment (**Figure 1A**). Diabetic mice displayed increased urine albumin to creatinine ratios (uACR), an indicator of kidney damage which was assessed at 12 weeks after diabetic establishment (**Figure 1B**). Western blot analysis showed that the protein of MAD2B was upregulated in isolated glomeruli of DN mice compared with the nondiabetic group (**Figure 1C-D**). A similar trend was observed for MAD2B expression in the glomeruli of renal biopsies (**Figure 1E-F**).* In vitro*, osmotic pressure did not affect the expression of MAD2B in podocytes with mannitol stimulating (**Figure 1G-H**). However, MAD2B was continuously increasing with the prolonged stimulation of high glucose, a common detrimental factor in diabetics (**Figure 1I-J**). The above results suggested that MAD2B might play a potentially important role in the STZ-induced diabetic nephropathy.

### Construction of conditional gene knockout mice with podocyte-specific deletion of MAD2B (Cre^+^/MAD2B^fl/fl^)

**Figure 2:**
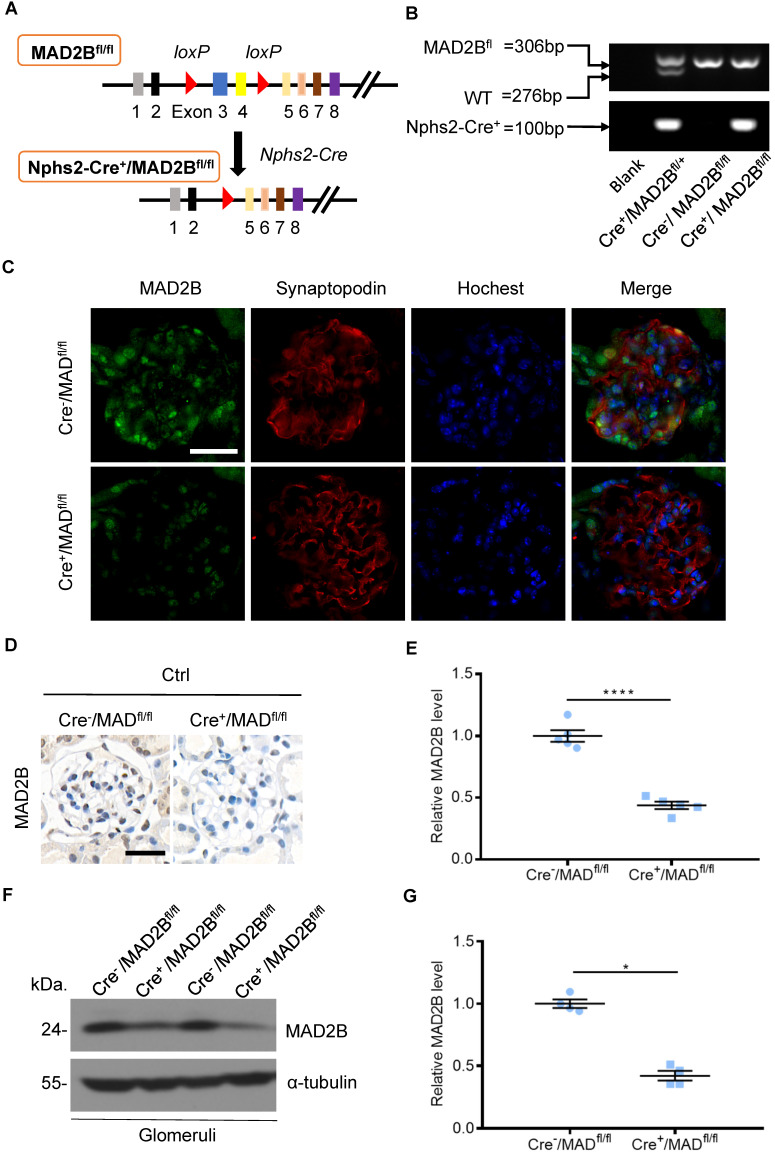

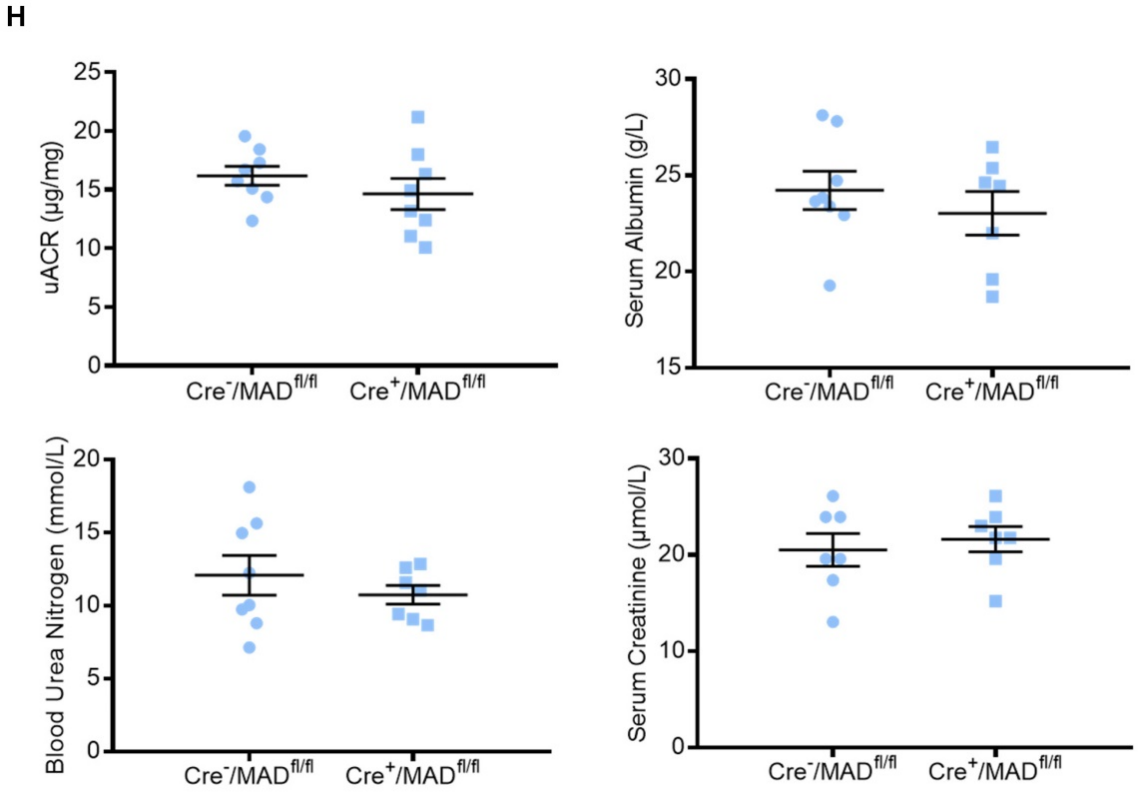
** Construction of conditional gene knockout mice with podocyte-specific deletion of MAD2B (Cre^+^/MAD2B^fl/fl^). (A)** Construction of Cre^+^/MAD2B^fl/fl^ mice by using Cre-LoxP recombination system. Exon 3 and 4 are deleted upon Nphs2-Cre mediated recombination. **(B)** Genotyping was confirmed by tail preparation and PCR at 4 weeks of age. **(C)** Representative IF imaging of MAD2B (green) and synaptopodin (red) in glomeruli from Cre^-^/MAD2B^fl/fl^ and Cre^+^/MAD2B^fl/fl^ mice (n=4). Scale bar: 25 µm. **(D and E)** Representative immunohistochemical images and quantification of glomerular MAD2B expression in Cre^-^/MAD2B^fl/fl^ and Cre^+^/MAD2B^fl/fl^ mice (n = 5). Scale bar: 25 µm. ****P<0.0001. **(F and G)** Representative western blot and quantification of MAD2B expression in isolated glomeruli from Cre^-^/MAD2B^fl/fl^ and Cre^+^/MAD2B^fl/fl^ mice (n = 4). *P<0.05 vs Cre^-^/MAD2B^fl/fl^. **(H)** There was no difference in the serum albumin, urine albumin to creatinine ratio (uACR), blood urea nitrogen and serum creatinine between Cre^-^/MAD2B^fl/fl^ and Cre^+^ /MAD2B^fl/fl^ mice (n = 7-8). *P*>0.05 vs Cre^-^/MAD2B^fl/fl^. All data were expressed as mean ± SEM.

To determine the role of endogenous MAD2B in the progression of DN, podocyte-specific MAD2B knockout (Cre^+^/MAD2B^fl/fl^) mice were generated by a Cre-Loxp recombination system (**Figure 2A**), which was confirmed by tail genotyping (**Figure 2B**). Immunofluorescent results showed significant reduced MAD2B expression in the glomeruli co-located with synaptopodin (**Figure 2C**), a podocyte marker protein. Moreover, we obtained similar MAD2B reduction in the glomeruli of Cre^+^/MAD2B^fl/fl^ mice compared to Cre^-^/MAD2B^fl/fl^ mice by immunohistochemical (**Figure 2D-E**) and western blot analyses (**Figure 2F-G**). Meanwhile, we found that there was no significant difference in serum albumin, uACR, blood urea nitrogen (BUN) and serum creatinine (SCr) between Cre^-^/MAD2B^fl/fl^ and Cre^+^/MAD2B^fl/fl^ mice (**Figure 2H**). These data meant that podocyte-specific MAD2B knockout mice were successfully constructed.

### Podocyte-specific MAD2B deletion alleviated renal injury in STZ-induced DN mice

**Figure 3:**
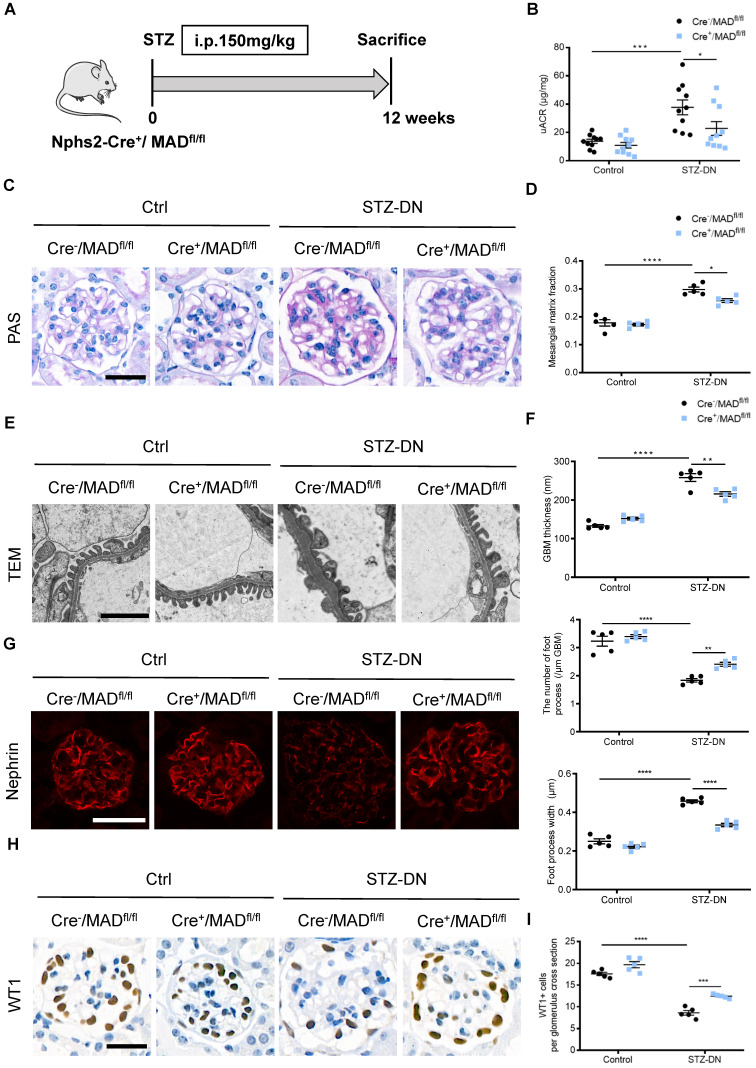
** Podocyte-specific MAD2B deletion alleviated renal injury in STZ-induced DN mice. (A)** A schematic diagram showing the method of building STZ-induced DN model. Eight weeks old Cre^+^/MAD2B^fl/fl^ mice were given intraperitoneal injection of 150 mg/kg streptozotocin and sacrificed after 12 weeks. **(B)** Urine albumin to creatinine ratio (uACR) in mice (n = 10). *P<0.05, ***P<0.001. **(C)** Periodic acid-Schiff (PAS) staining showing glomerular morphological changes. Scale bar: 25 µm. **(D)** Quantitative analyses of the percentage of mesangial matrix area (n = 5). *P<0.05, ****P<0.0001. **(E)** Representative transmission electron microscopy (TEM) images showing morphological changes in the podocyte foot process in different groups of mice. Scale bar: 2 µm. **(F)** Indices for glomerular filtration barrier integrity, including glomerular basement membrane (GBM) thickness, foot process width, and the number of foot processes/µm GBM (n = 5). **P<0.01, ****P<0.0001. **(G)** Representative images of Nephrin in glomeruli from control and STZ-induced mice. Scale bar: 25 µm. **(H and I)** Representative images of Wilms' Tumor 1 (WT1) and quantifications of WT1-positive cells per glomerulus in kidney sections (n = 5). Scale bar: 25 µm. ***P<0.001, ****P<0.0001. All data were expressed as mean ± SEM.

DN model was induced in Cre^-^/MAD2B^fl/fl^ mice and Cre^+^/MAD2B^fl/fl^ mice by single high-dose intraperitoneal injection of STZ (**Figure 3A**). Diabetic Cre^+^/MAD2B^fl/fl^ mice showed lower urinary albumin excretion compared to diabetic Cre^-^/MAD2B^fl/fl^ mice (**Figure 3B**). Consistent with improved kidney function, mesangial matrix expansion was less severe in diabetic Cre^+^/MAD2B^fl/fl^ mice than Cre^-^/MAD2B^fl/fl^ mice (**Figure 3C-D**). Electron microscopy showed mitigated glomerular basement membrane (GBM) thickening and podocyte foot process effacement in diabetic Cre^+^/MAD2B^fl/fl^ mice, along with foot process width reduction (**Figure 3E-F**). In addition, podocyte-specific MAD2B deletion in mice reversed the diabetes-induced decline of Nephrin (a podocyte marker protein) expression (**Figure 3G**) and loss of podocytes by observing WT 1-positive cells (**Figure 3H-I**). The above results suggest that increased MAD2B expression contribute to diabetic related podocyte injury which can be reversed by podocyte-specific MAD2B deletion.

### MAD2B interacted and co-localized with Numb in mice kidney and cultured podocytes

**Figure 4:**
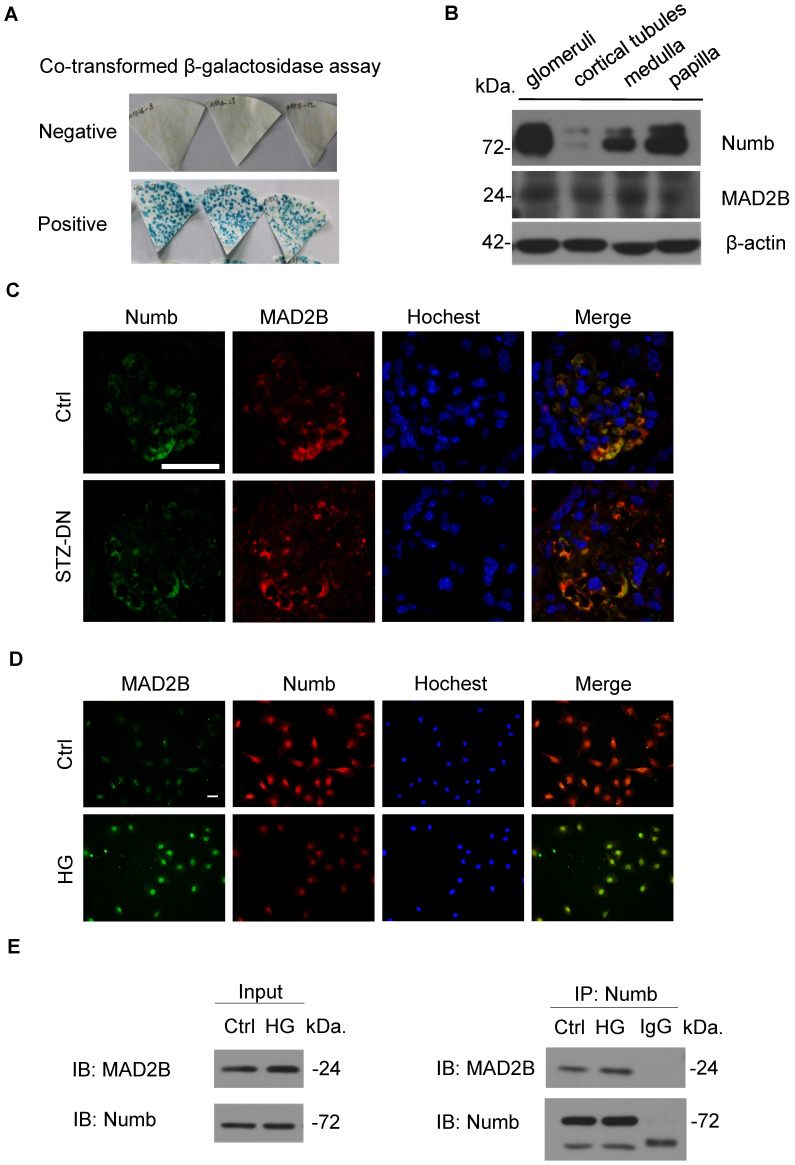
** Co-localization and endogenous interaction between MAD2B and Numb in mice kidney and cultured podocytes. (A)** Yeast two-hybrid screening realizing that Numb was one of the MAD2B binding proteins. **(B)** Representative western blot images showing the expression of Numb and MAD2B in different compartments of mice kidney (n = 3). Glomeruli and cortical tubules were isolated by Dynabead perfusion from mouse. **(C)** Representative IF imaging of Numb (green) and MAD2B (red) in glomeruli from control and DN mice (n = 3). Scale bar: 25 µm. **(D)** IF staining and co-localization of MAD2B (green) and Numb (red) in human podocytes (HPCs) (Original magnification: ×200). Scale bar: 25 µm. **(E)** Whole protein lysates of podocytes treated with HG was collected and immunoprecipitated (IP) with anti-Numb or IgG followed by western blot analysis (IB) with an anti-MAD2B antibody and anti-Numb antibody.

To explore the specific mechanism of MAD2B on podocyte injury of DN, we performed a yeast two-hybrid analysis confirming the endogenous interaction between MAD2B and Numb (**Figure 4A**). Meanwhile, we characterized the expression of Numb and MAD2B in normal C57BL/6J mice renal tissues by western blot which showed that both of them were well expressed in mice glomeruli (**Figure 4B**). IF staining proved the colocalization of MAD2B and Numb in diabetic mice glomeruli (**Figure 4C**) and podocytes under HG conditions (**Figure 4D**). Moreover, co-immunoprecipitation of MAD2B and Numb in human podocytes (HPCs) demonstrated that MAD2B physically interacted with Numb (**Figure 4E**). In conclusion, MAD2B interacted with Numb both *in vitro* and *in vivo*.

### Numb was downregulated in podocytes under high glucose condition and in the kidneys from STZ-induced diabetic mice

**Figure 5:**
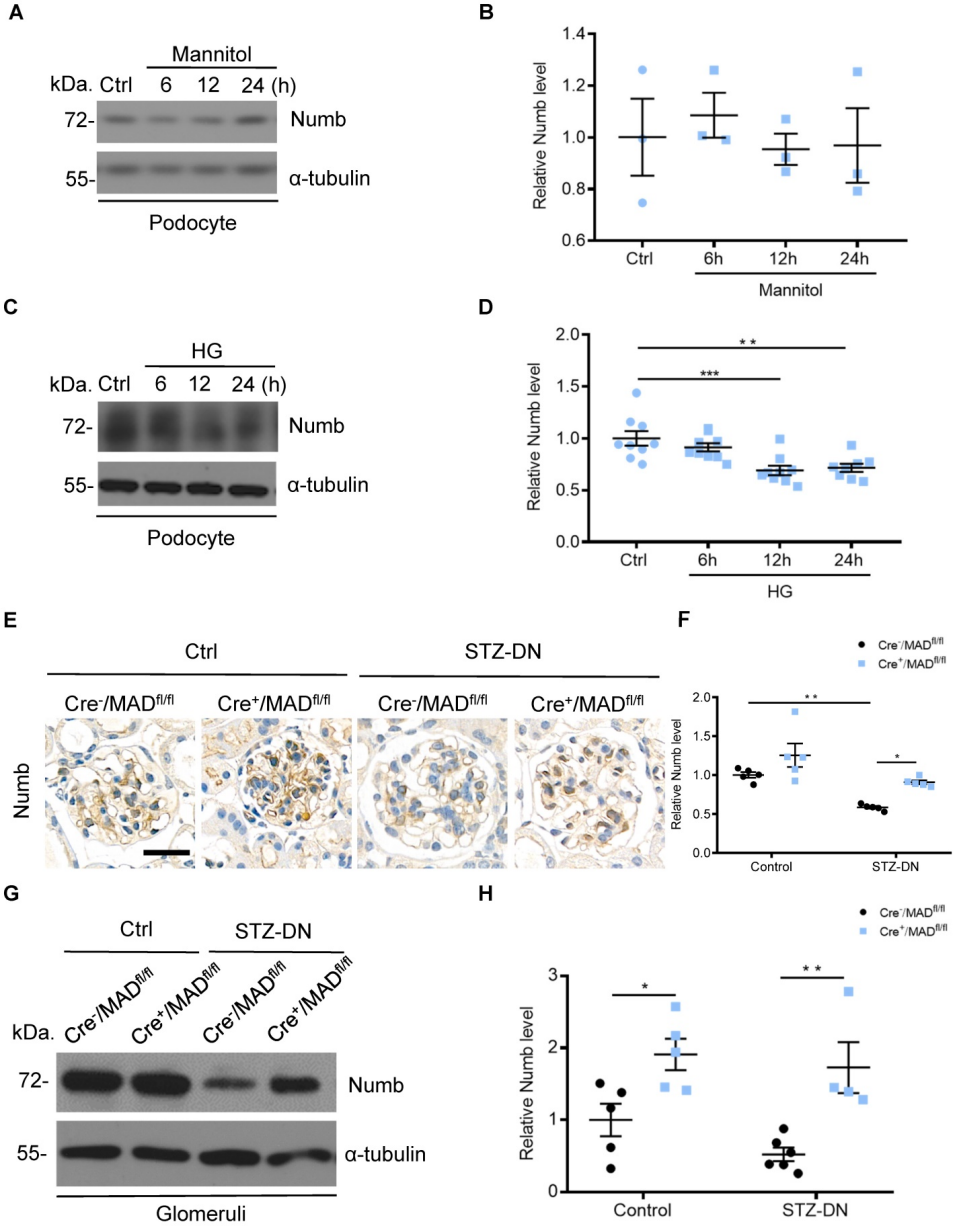
** Numb was downregulated in podocytes under high glucose condition and in the kidney from STZ-induced diabetic mice. (A and B)** Representative western blot and quantification data presenting the relative Numb protein levels in podocytes exposed to 29.4 mM mannitol plus 5.6 mmol/l D-glucose for indicated time. (n = 3), *P*>0.05. **(C and D)** Representative western blot and quantification of Numb expression in podocytes exposed to 35mM HG for indicated time (n= 8-9). **P<0.01, ***P<0.001. **(E and F)** Representative immunohistochemical images and quantification of glomerular Numb expression in STZ-induced diabetic mice (n = 5). Scale bar: 25 µm. *P<0.05, **P<0.01. **(G and H)** Representative western blot and quantification of Numb expression in isolated glomeruli from diabetic Cre^-^/MAD2B^fl/fl^ and Cre^+^/MAD2B^fl/fl^ mice (n = 4-6). *P<0.05, **P<0.01. All data were expressed as mean ± SEM.

Considering that MAD2B and Numb were closely related, we then detected Numb expression in podocytes. Western blot analysis revealed that the expression of Numb showed no significant change under mannitol stimulation (**Figure 5A-B**), but it was continuously declining with the prolonged stimulation of HG (**Figure 5C-D**). *In vivo,* Cre^-^/MAD2B^fl/fl^ mice and Cre^+^/MAD2B^fl/fl^ mice were induced to develop DN with STZ treatment. A similar significant decrease in the expression of Numb was observed in the glomeruli of diabetic Cre^-^/MAD2B^fl/fl^ mice compared to the control Cre^-^/MAD2B^fl/fl^ mice, while the downward trend was reversed in Cre^+^/MAD2B^fl/fl^ mice by IHC (**Figure 5E-F**) and western blot (**Figure 5G-H**). These findings suggested that Numb was involved in MAD2B-mediated regulatory mechanisms in the DN model.

### MAD2B silencing reversed the decline of Numb in podocytes exposed to HG and thus regulated Numb-Notch1 pathway *in vitro* and *in vivo*

**Figure 6:**
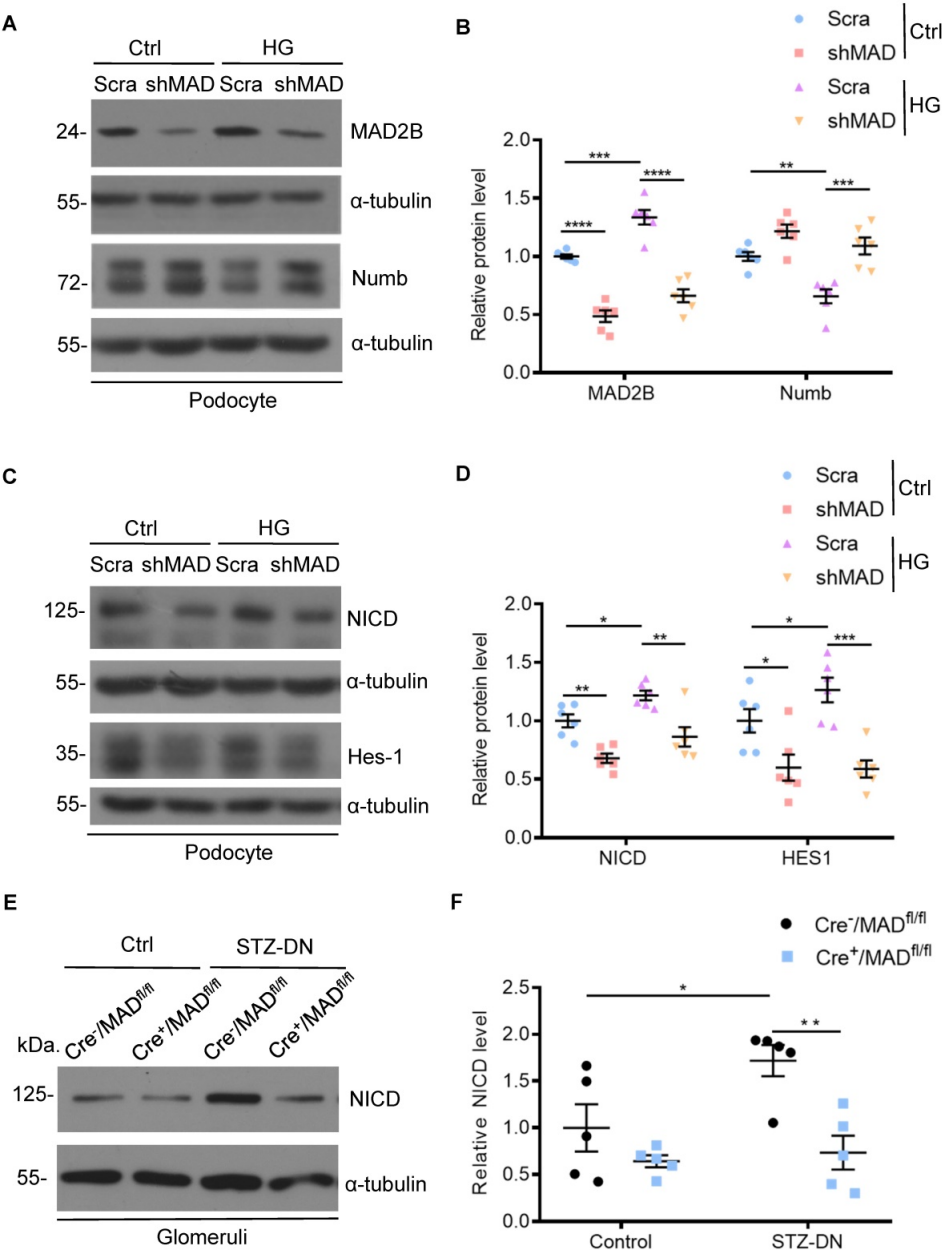
** MAD2B silencing reversed the decline of Numb in podocytes when exposed to HG and thus regulated Numb-Notch1 pathway *in vitro* and *in vivo*.** Cells were transfected with MAD2B shRNA or scramble shRNA and then exposed to 35 mM HG for 24 h. **(A and B)** Representative western blot images and quantification data showing that MAD2B was decreased when transfected with MAD2B shRNA while Numb expression was increased. (n = 6). **(C and D)** Representative western blot images and quantification data showing that MAD2B downregulation depressed NICD and HES-1 expression which are downstream proteins of Numb (n = 6). **(E and F)** Representative western blot images and quantification of NICD in the control and STZ-induced glomeruli of Cre^-^/MAD2B^fl/fl^ and Cre^+^/MAD2B^fl/fl^ mice (n = 5). Scra: scrambled shRNA; shMAD: MAD2B shRNA. *P<0.05, **P<0.01, ***P<0.001, ****P<0.0001. All data were expressed as mean ± SEM.

Having established that Numb correlated negatively with MAD2B in podocytes under HG environment and in diabetic mice, we tried to clarify the underlying relationship. First, we silenced MAD2B using shRNA lentivirus *in vitro* to clarify whether reduced MAD2B expression affects Numb expression in podocytes. As shown in **Figure 6A-B**, HG-induced reduction of Numb expression in podocytes was significantly elevated by the deletion of MAD2B. On the contrary, the classical downstream signaling of Numb, NICD and Hes-1 expressions substantially decreased with MAD2B transfection (**Figure 6C-D**). Similarly, the expression of NICD was significantly declined in diabetic Cre^+^/MAD2B^fl/fl^ mice glomeruli compared to diabetic Cre^-^/MAD2B^fl/fl^ mice (**Figure 6E-F**). Hence, these evidences indicated that, as the expression of MAD2B decreased, Numb expression increased significantly, resulting in the attenuation of Notch 1 signaling.

### Overexpression of Numb alleviated podocyte injury

**Figure 7:**
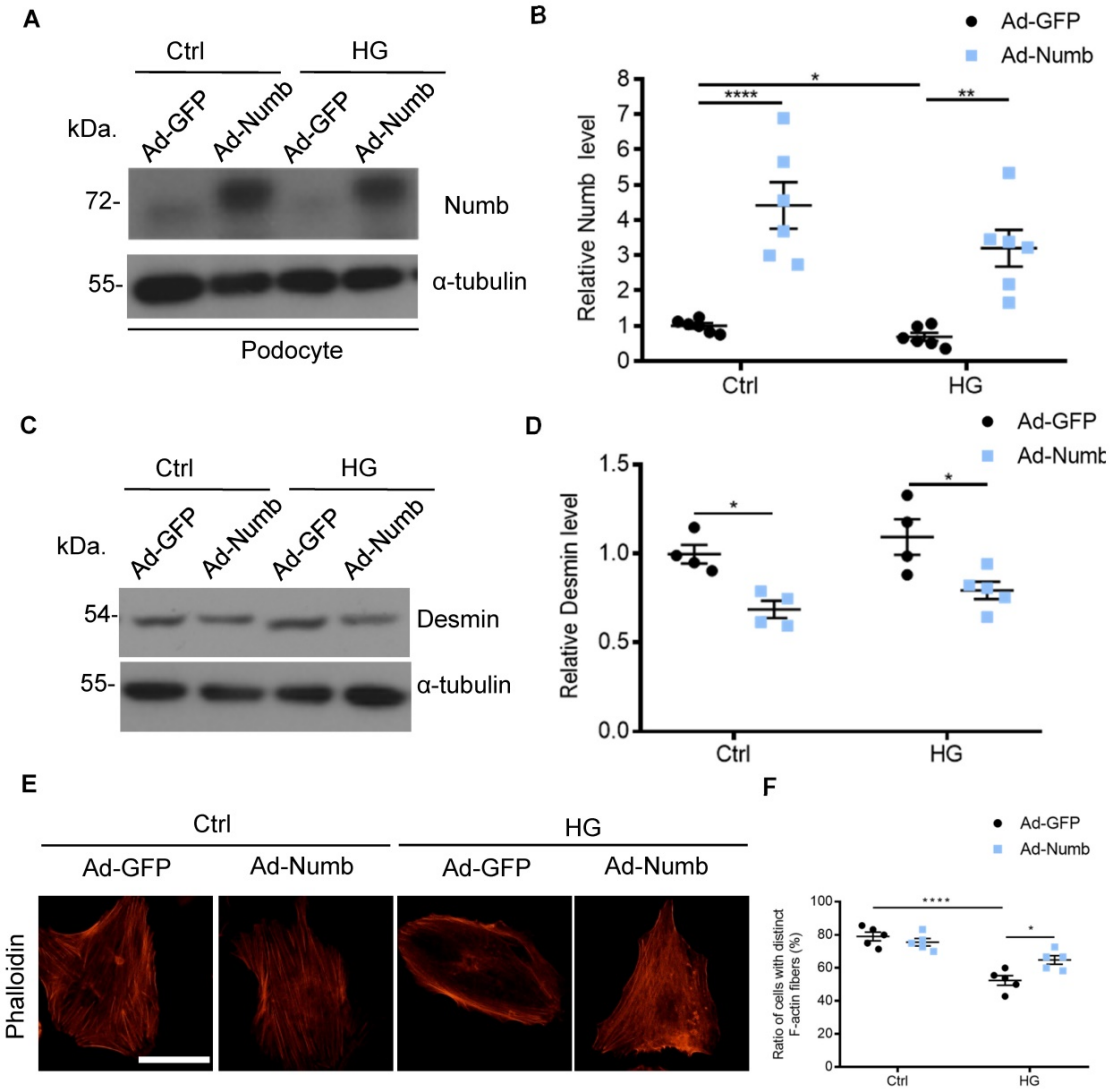
** Overexpression of Numb alleviated podocyte injury.** Cells were transfected with green fluorescent protein (GFP) adenovirus (Ad-GFP) or Numb adenovirous (Ad-Numb) and then exposed 35 mM HG for 24 h. **(A and B)** Representative western blot images and quantification data displaying the relative Numb protein level (n = 6). **(C and D)** Representative western blot images and quantification data showing that Desmin expression was decreased by Numb overexpression (n = 4). **(E)** Microscopic images of F-actin by rhodamine-phalloidin staining. Scale bar: 50 µm. **(F)** Summarized data from counting the cells with distinct, longitudinal F-actin fibers. Scoring was determined from 100 podocytes on each slide (n = 5). *P<0.05, **P<0.01, ****P<0.0001. All data were expressed as mean ± SEM.

Accumulated evidence pointed to the association of MAD2B and Numb in DN. To determine whether decreased Numb expression contributes to podocyte injury in DN, we applied Numb overexpression adenovirus (Ad-Numb) and control GFP adenovirus (Ad-GFP) to construct a cell model (**Figure 7A-B**). We examined Desmin expression under HG-stimulated condition with or without Numb overexpression. As shown in **Figure 7C-D**, Numb overexpression reversed the elevation of Desmin expression induced by HG. In addition, podocyte cytoskeleton disarrangement was gradually relieved with Numb transfection (**Figure 7E-F**). Above all, we suggested that Numb mediated protective effect on podocytes under diabetic conditions.

## Discussion

Although our previous studies have indicated the pathogenic effects of MAD2B on tubulointerstitial fibrosis, crescentic glomerulonephritis and podocyte injury 9, 23, 24, deeper understanding of biological functions of MAD2B in diabetic nephropathy is very limited and the precise mechanisms how MAD2B regulates podocyte injury remain to be elucidated. Our study was designed to further study the functional role of MAD2B in the kidney. In this study, we have revealed that MAD2B contributes to podocyte injury in DN. The potential mechanism is that MAD2B binds to Numb, promotes its degradation and inhibits its negative regulatory effect on Notch 1 signaling, resulting in sustained Notch 1 signaling activation and the subsequently podocyte injury in DN.

First, we demonstrated that MAD2B was upregulated in the glomeruli from STZ-induced DN mice and in podocytes under high glucose condition. In order to explore the role of MAD2B on the regulation of podocyte function, we established podocyte-specific MAD2B knockout (Cre^+^/MAD2B^fl/fl^) mice and the Cre^+^/MAD2B^fl/fl^ mice did not exhibit significant proteinuria or glomerular injury compared with the Cre^-^/MAD2B^fl/fl^ mice. This finding supports the reliability of the results in knockout mice. To explore the role of MAD2B in DN, we established an STZ-induced diabetic model in MAD2B knockout mice. The increase of urinary albumin excretion and the severity of podocyte and glomerular injury in Cre^+^/MAD2B^fl/fl^ diabetic mice were significantly less than Cre^-^/MAD2B^fl/fl^ diabetic mice, indicating that podocyte-specific MAD2B deletion alleviates podocyte injury in DN. Namely, upregulated MAD2B promotes podocyte injury in DN, further studies are required to identify the related underlying mechanism.

Through yeast two-hybrid analysis, we found the endogenous interaction between MAD2B and Numb. Numb is originally identified as an intrinsic cell fate determinant since it interacts with various cell cycle-related molecules of different singling pathways, including Notch, Hedgehog and P53 25. In the kidney, Numb has been shown to play a protective role in cisplatin-induced acute kidney injury (AKI) through ameliorating tubular necrosis and renal inflammation 26, but it can also aggravate progressive tubulointerstitial fibrosis (TIF) by promoting G2/M arrest 27. Emerging evidence has indicated that Numb acts as a double-edged sword in the renal tubular disease, which protects human renal tubular epithelial cells (HKCs) from BSA-induced apoptosis and aggravates renal fibrosis by promoting tubular epithelial cell cycle arrest at G2/M 28,29. However, few studies were carried out to investigate the role of Numb in podocyte injury and in diabetic nephropathy. In this study, we confirmed for the first time that Numb was downregulated in HG-stimulated podocytes and in glomeruli of STZ-induced DN mice. Whereas the decline of Numb expression could be reversed by knocking down MAD2B *in vitro* and *in vivo*. In addition, we induced Numb overexpression in podocyte and found that Numb protected podocytes by maintaining the actin cytoskeleton. These data suggest the down-regulation of Numb induced by diabetic factors contributes to podocyte injury in DN progression, further studies are needed to probe possible mechanisms.

Considering that activation of Notch 1 signaling pathway results in podocyte damage and depletion in the glomeruli of diabetic mice 30,31, and Numb suppresses Notch 1 signaling pathway by binding with Notch 1 intercellular domain (NICD), leading to the ubiquitination and degradation of NICD 32, Notch 1 may be a potential target of MAD2B. In mammals, the Notch pathway consists of four receptors (Notch 1∼4) and five ligands including delta-like (DLL) 1, DLL-3, DLL-4, Jagged1 and Jagged2 33. Studies have demonstrated that podocyte-specific re-expression of NICD in mice caused severe proteinuria and podocyte apoptosis, resulting in glomerulosclerosis 34. In this study, we discovered a novel role of MAD2B in Notch1 signaling. Silencing MAD2B reduced the expression of NICD in HG-treated podocyte, which was consistent with the result that the expression of NICD was significantly decreased in podocytes from Cre^+^/MAD2B^fl/fl^ mice with DN. We further found that the level of Hes-1, Notch downstream target gene 35, declined as well as NICD after knockdown of MAD2B in podocyte. Above all, we suggest that MAD2B promotes podocyte injury by regulating Numb-dependent Notch1 pathway in DN.

However, the detailed interactions and regulatory mechanisms between MAD2B and Numb still need to be clarified. It has been demonstrated that APC2, a scaffold subunit of APC/C, binds to a ubiquitination enzyme—MDM2 (murine double minute 2). APC2 is reported to be required for MDM2 polyubiquitination and proteasomal degradation in mouse embryonic fibroblasts, and depletion of APC2 results in increased levels of MDM2 36. In addition, it has been confirmed that Numb is ubiquitinated by MDM2 37. Thus, it is possible that the upregulation of MAD2B under hyperglycemic condition may inhibit APC/C activation, resulting in the accumulation of MDM2, which leads to the ubiquitination and degradation of Numb. Furthermore, we have discovered that high glucose up-regulates MDM2 expression in podocyte 18 and MAD2B deletion leads to decreased expression of MDM2 in podocytes under hyperglycemic condition (**Figure S1**). The above evidence is consistent with our hypothesis.

In this study, we cannot exclude other Notch receptors (Notch 2-4) may also contribute to regulating podocyte function since emerging evidence have verified re-expression of Notch 2-4 in podocyte of mice under pathological conditions including focal segmental glomerulosclerosis (FSGS), rapid progressive glomerulonephritis (RPGN) and DN 12, 38, 39. Besides Notch receptors, other signaling pathways regulated by Numb, such as Wnt 40, may contribute to podocyte injury as well. Wnt/β-catenin signaling was proved to promote podocyte dysfunction and albuminuria in DN 41, but our study suggested that MAD2B deletion didn't affect Wnt/β-catenin pathway *in vitro* and *in vivo* (**Figure S2**). Therefore, it is necessary to further clarify other Notch receptors and other downstream targets potentially regulated by MAD2B in podocytes.

**Figure 8:**
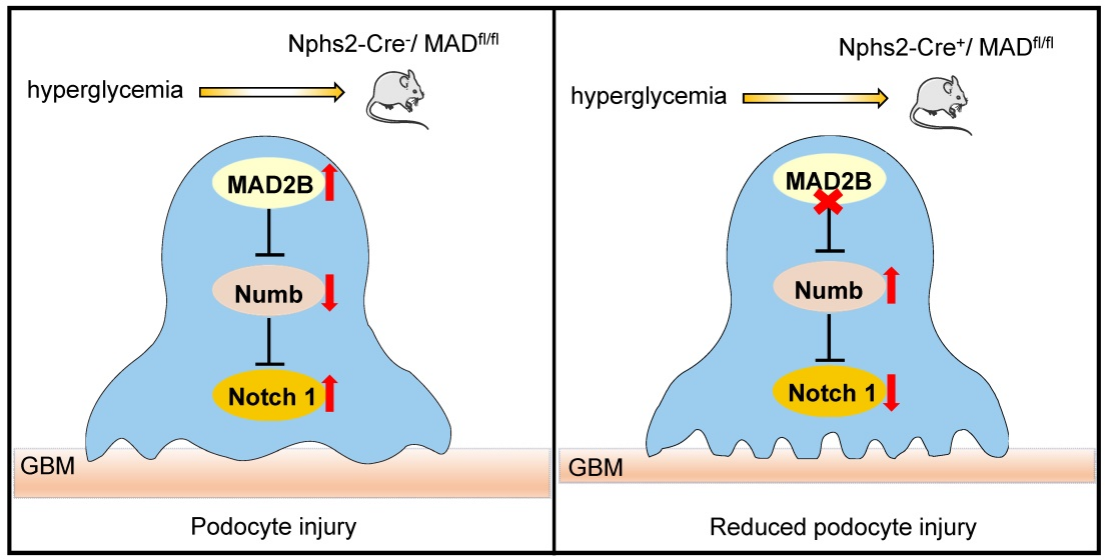
** Proposed model illustrating MAD2B deficiency alleviates podocyte injury through regulating Numb-dependent Notch1 pathway in diabetic nephropathy.** Under hyperglycemic conditions, upregulation of MAD2B enhances the activity of Notch 1 signaling by decreasing the expression of Numb, which finally results in podocyte injury. Podocyte-specific MAD2B deletion leads to the increased expression of Numb, thereby inhibiting Notch 1 signaling and alleviating podocyte injury.

In summary, our study demonstrates that MAD2B promotes podocyte injury in DN, through inhibiting the role of Numb in negatively regulating Notch1 signaling. Although the detailed interactions and regulatory mechanisms between MAD2B and Numb still need to be clarified, therapeutic approaches to specifically modulate MAD2B-mediated Numb-dependent Notch1 pathway in the kidneys may prevent or halt the progression of DN (**Figure 8**).

## Supplementary Material

Supplementary figures.

## Abbreviations

DN: diabetic nephropathy
HG: high glucose
STZ: streptozotocin
ESRD: end-stage renal disease
GFR: glomerular filtration rate
MAD2B: mitotic spindle assembly check point protein 2
APC/C: anaphase-promoting complex/cyclosome
EMT: epithelial-mesenchymal transition
RGEC: human glomerular endothelial cells
uACR: urine albumin to creatinine ratio
BUN: blood urea nitrogen
SCr: serum creatinine
GBM: glomerular basement membrane
HPCs: human podocytes
AKI: acute kidney injury
TIF: tubulointerstitial fibrosis
HKCs: human renal tubular epithelial cells
NICD: Notch 1 intercellular domain
RPGN: rapid progressive glomerulonephritis
MDM2: murine double minute 2
IF: immunofluorescence staining
IHC: immunohistochemistry
Co-IP: co-immunoprecipitation
