# Supplementary material for: MAD2B promotes podocyte injury through regulating Numb-dependent Notch 1 pathway in diabetic nephropathy
Source: Int J Biol Sci. 2022 Feb 21;18(5):1896–911. doi: 10.7150/ijbs.68977 (PMC8935242; doi:10.7150/ijbs.68977)
Supplement: Supplementary file 1 — Supplementary figures. [file ijbsv18p1896s1.pdf]

## Supplementary Figures and Figure Legends

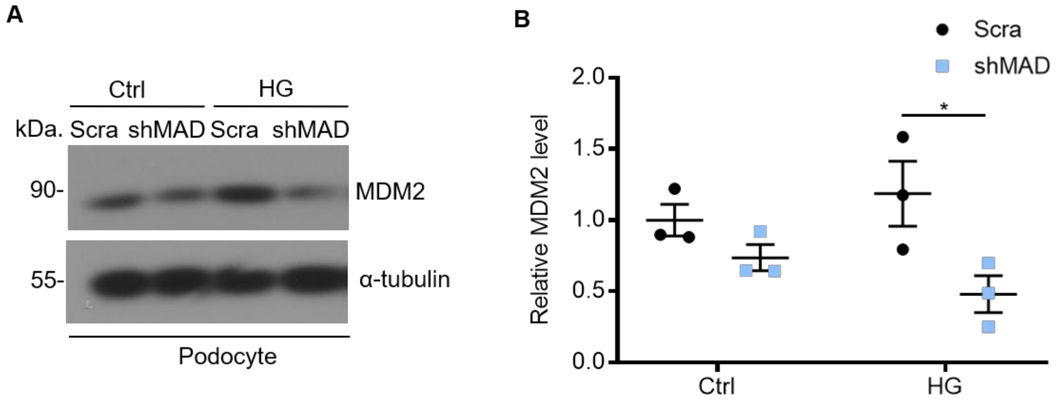


**Figure S1. MAD2B silencing led to decreased expression of MDM2 in podocytes when exposed to HG.** Cells were transfected with MAD2B shRNA or scramble shRNA and then exposed to 35 mM HG for 24 h. (A) Representative western blot images of MDM2 in HG-stimulated podocytes when transfected with MAD2B shRNA. (n = 3). (B) Corresponding quantifications of MDM2. (n = 3). Scra: scrambled shRNA; shMAD: MAD2B shRNA. **P<0.05*. All data were expressed as mean ± SEM.

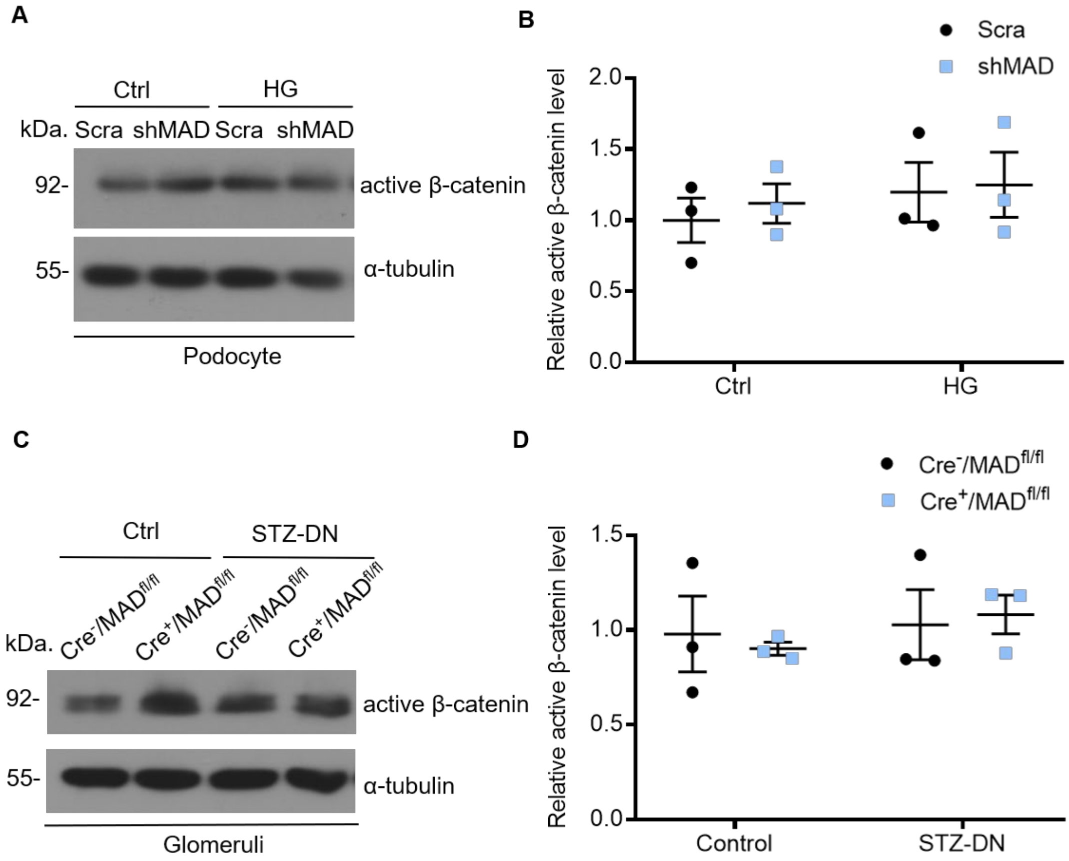


**Figure S2. MAD2B silencing had no effect on Wnt/β-catenin pathway in vitro and in vivo.** Cells were transfected with MAD2B shRNA or scramble shRNA and then exposed to 35 mM HG for 24 h. (A and B) Representative western blot images and quantification data showing that there was no significant difference in the expression of active β-catenin between different groups. (n = 3). (C and D) Representative western blot images and quantification of active β-catenin in the control and STZ-induced glomeruli of $Cre^-/MAD2B^{fl/fl}$ and $Cre^+/MAD2B^{fl/fl}$ mice (n = 3). Scra: scrambled shRNA; shMAD: MAD2B shRNA. P>0.05. All data were expressed as mean ± SEM.
